# Sarcoid-like reaction induced by neoadjuvant immunotherapy in Stage III non-small cell lung cancer

**DOI:** 10.31744/einstein_journal/2024AI0810

**Published:** 2024-03-19

**Authors:** Leonardo Chaves Machado, Eduardo Kaiser Ururahy Nunes Fonseca, Genival Viana de Oliveira, Gustavo Schvartsman, Rodrigo Caruso Chate

**Affiliations:** 1 Hospital Israelita Albert Einstein Department of Radiology and Imaging Diagnosis São Paulo SP Brazil Department of Radiology and Imaging Diagnosis, Hospital Israelita Albert Einstein, São Paulo, SP, Brazil.; 2 Hospital Israelita Albert Einstein Department of Oncology São Paulo SP Brazil Department of Oncology, Hospital Israelita Albert Einstein, São Paulo, SP, Brazil.

**Figure 1:**
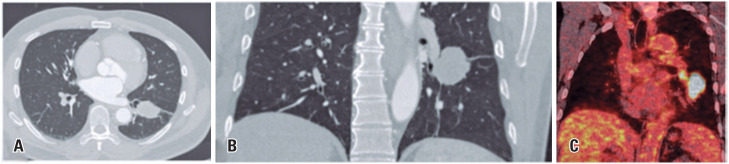
Coronal and axial views of coronary angiotomography with pulmonary window (A and B) revealed a lung mass located in the left lower lobe. The mass showed contact with the origin of the basal segment bronchi, resulting in mild subsegmental bronchi obliteration. Furthermore, the PET-CT scan reformatted image (C) showed intense glycolytic hypermetabolism (SUVmax 26.5) within this lesion, indicating a high likelihood of a primary neoplasm, and a suspected lymph node (SUVmax 6.9) involvement

**Figure 2:**
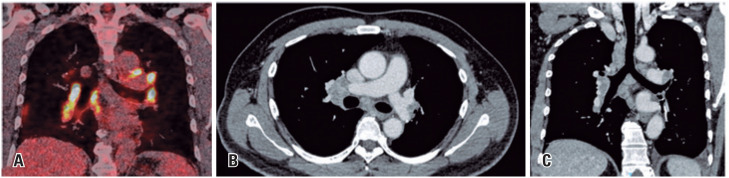
Following the initiation of treatment, the coronal 2-deoxy-2-[fluorine-18] fluoro- D-glucose ^18^F-FDG)-PET/CT view (A) revealed primary tumor and secondary lymph node response but also enlarged lymph nodes with increased FDG uptake (SUVmax 36.9) in the mediastinal chain and pulmonary hila. Subsequent post-operative axial and coronal chest CT images with mediastinum window settings (B and C) demonstrated multiple enlarged mediastinal and hilar lymph nodes with heterogeneous enhancement. In this clinical context, the possibility of sarcoid-like reactions induced by immunotherapy was suspected

Lung cancer represents the most commonly diagnosed neoplasm and the leading cause of cancer-related death worldwide, with majority of the patients being histologically categorized as non-small cell lung cancer. We present the case of a 54-year-old male patient, with a smoking history of 30 pack-years and no other comorbidities, with a pulmonary mass in the left lower lobe (Figure 1) incidentally revealed by computed tomography (CT). After pathological confirmation of adenocarcinoma with hilar lymph node metastasis (cT2b pN1 M0, EIIB), the patient underwent neoadjuvant therapy with carboplatin AUC6, pemetrexed 500mg/m^2^, and nivolumab 360mg (three cycles with a three-week interval), with a planned curative lower left lobectomy and lymph node dissection. Positron emission tomography (PET)-CT images following neoadjuvant treatment revealed increased size and heterogeneous enhancement of bilateral mediastinal and hilar lymph nodes (Figure 2). Concerns about disease progression were raised, but histological analysis indicated a granulomatous reaction consistent with a sarcoid-like reaction (SLR), with post-neoadjuvant pathological staging of ypT1c ypN0.

When considering oncological treatment, an SLR refers to a non-caseating granulomatous reaction observed in patients receiving active treatment.^(1-3)^ Sarcoid-like reactions have been associated with various drugs, including anti-programmed cell death protein 1 (PD-1) inhibitors like nivolumab.^(1)^ This drug triggers an antitumor response, slows tumor growth, and promotes tumor rejection by stimulating memory antigen-specific T cell proliferation.^(4)^ The mechanism underlying SLR remains unclear; however, it has been postulated that PD-1 blockade enhances interferon-gamma release, which may reactivate previous immune responses.^(1,3,5)^

Sarcoid-like reactions are important considerations in patients undergoing oncological treatment because they can mimic disease progression.^(1)^ Women appear to be predisposed to SLR, associated with multiple myeloma and non-small cell lung cancer.^(1)^ Mediastinal and hilar lymph node enlargements are the most common imaging findings.^(1,2)^ Parenchymal pulmonary changes have also been reported, including septal thickening and subpleural/fissural nodules;^(1)^ however, these findings were not observed in the present case. Pleural effusion aids in the differential diagnosis and is typically absent in classic sarcoidosis.^(1)^ Although no specific treatment exists, discontinuation of immunotherapy often leads to a reduction in reaction severity.^(1)^ Accurate diagnosis of SLR relies on histopathological analysis, which may typically reveal sarcoid-like granulomas.^(1)^

This case highlights the importance of recognizing SLR as a potential diagnosis in patients undergoing active anti-cancer therapy, which can be confounded by disease progression. Understanding the association between SLR and cancer immunotherapy is crucial for patient management and avoiding unnecessary interventions.
